# Dedicated Robotic‐Assisted Microsurgery Platforms in Plastic and Reconstructive Surgery: A Systematic Review

**DOI:** 10.1002/micr.70286

**Published:** 2026-08-27

**Authors:** Simon M. Kratzer, Mehmet Ergisi, Laura Awad, Deepak M. Kalaskar, Peter Butler, Dariush Nikkhah

**Affiliations:** ^1^ Division of Surgery & Interventional Science, Royal Free London NHS Foundation Trust University College London London UK; ^2^ Department of Plastic and Reconstructive Surgery, Royal Free London NHS Foundation Trust, and Division of Surgery and Interventional Science University College London London UK

**Keywords:** free flap reconstruction, learning curve dynamics, plastic and reconstructive surgery, robotic‐assisted microsurgery (RAMS), supermicrosurgery, Symani

## Abstract

**Background:**

Dedicated robotic‐assisted microsurgical systems (RAMS), including the Symani Surgical System and MUSA, were developed to overcome physiological tremor, surgeon fatigue, and restricted maneuverability in deep surgical fields. Existing reviews of robotic plastic surgery largely span heterogeneous applications beyond microsurgery or lack structured risk‐of‐bias appraisal specific to dedicated microsurgical platforms. This review systematically synthesizes clinical outcomes, learning‐curve dynamics, and technological features of dedicated RAMS platforms in microsurgery, incorporating structured risk‐of‐bias appraisal to clarify the evidence base and inform clinical adoption.

**Methods:**

Studies published 2015–2025 were identified through PubMed, Embase, Scopus, and Web of Science (PROSPERO CRD420261320086). Following independent screening, 21 studies were included, assessing free flap survival, anastomotic patency, complications, operative times, and learning‐curve metrics.

**Results:**

Free flap reconstruction showed high survival (96%–100%); supermicrosurgical anastomosis patency was 96.6%–100%. Robotic anastomosis times fell 51%–66% with experience (largest reduction 66.1%, for deep‐plane arterial anastomosis), though times remained longer than manual benchmarks even after the learning phase (e.g., 25.3 ± 12.3 vs. 14.1 ± 4.3 min for mixed procedures). Conversion/failure was infrequent (0%–4.3%). Motion scaling (up to 20×) and tremor filtration were consistent advantages; haptic feedback remained universally absent.

**Conclusion:**

Dedicated RAMS platforms are safe and effective for micro‐ and supermicrosurgical reconstruction, non‐inferior to manual techniques. Initial time penalties resolve with experience. Future research should prioritize randomized controlled trials, standardized outcome measures, and cost‐effectiveness analyses.

## 1 Introduction

Reconstructive microsurgery has progressed from standard microvascular techniques to supermicrosurgery, where vessels under 0.8 mm are routinely addressed (Debeij et al. 2024). Even highly skilled microsurgeons remain constrained by human factors, namely physiological tremor, operative fatigue, and the ergonomic strain of prolonged static posture, which limit precision and demand extensive specialized training (Nikkhah et al. 2023).

Robotic‐assisted microsurgery (RAMS) emerged to address these limitations. Early attempts adapted general‐purpose platforms, notably the da Vinci Surgical System, which demonstrated feasibility for some microvascular tasks (Lee et al. 2012) but whose large footprint, laparoscopy‐oriented visualization, and instruments not optimized for 8–0 to 12–0 sutures precluded widespread adoption in dedicated microsurgery (Tan et al. 2018).

The transformative step has been dedicated RAMS platforms, the Symani Surgical System (Medical Microinstruments) and MUSA (Microsure), engineered specifically for micro‐ and supermicrosurgery with high motion scaling, tremor filtration, and ultra‐fine instruments. Clinical data have since accumulated rapidly across free tissue transfer, lymphatic reconstruction (LVA, VLNT), and peripheral nerve repair (von Reibnitz et al. 2024; Struebing et al. 2025; Aman et al. 2024).

While enthusiasm for this technology is high, the evidence remains fragmented. Justifying the capital and training investment for adoption demands a systematic understanding of clinical trade‐offs, not only success rates but also learning‐phase efficiency losses and ergonomic benefits to the surgeon.

Recent systematic and narrative reviews have begun to map robotic applications across plastic and reconstructive surgery more broadly (Ruccia et al. 2024; Kawashima et al. 2025; Brown et al. 2025). Reviews spanning breast, craniofacial, and aesthetic applications alongside microsurgery (Ruccia et al. 2024; Kawashima et al. 2025) dilute the microsurgery‐specific evidence, while a supermicrosurgery‐focused review was restricted to vessels < 0.8 mm without formal risk‐of‐bias appraisal (Brown et al. 2025). None combined structured, tool‐based risk‐of‐bias assessment (RoB‐2, ROBINS‐I, MINORS) with quantitative learning‐curve and manual‐comparator time extraction across the full spectrum of dedicated microsurgical applications, free flap reconstruction, supermicrosurgery, and nerve repair.

This review synthesizes current clinical evidence on dedicated RAMS platforms, characterizing (a) clinical outcomes across key reconstructive applications, (b) learning‐curve dynamics, and (c) the impact of technological features on microsurgical performance to guide informed adoption and future research.

## 2 Methods

This review followed PRISMA 2020 guidelines and was prospectively registered with PROSPERO (Registration No. CRD420261320086). Scope was restricted to clinical studies of dedicated robotic systems for micro‐ and supermicrosurgery, predominantly the Symani Surgical System and MUSA robot.

### 2.1 Search Strategy and Study Selection

A systematic search of PubMed/MEDLINE, Embase, Scopus, and Web of Science was executed to identify relevant literature published between January 2015 and October 2025. The core search terms combined MeSH and free‐text terms:

*(“robotic microsurgery” OR “robot‐assisted microsurgery” OR “robotic‐assisted microsurgery” OR “microsurgical robot” OR “microsurgical system” OR “Symani” OR “MUSA” OR “da Vinci”) AND (“plastic surgery” OR “reconstructive surgery” OR “microsurgical reconstruction” OR “flap surgery” OR “lymphatic surgery” OR “nerve repair”) AND (“clinical outcomes” OR “operative time” OR “learning curve” OR “ergonomics” OR “feasibility” OR “precision” OR “cost‐effectiveness” OR “translation” OR “adoption” OR “implementation”)*.

The initial search yielded 318 records: 103 in PubMed/MEDLINE, 10 in Embase, 133 in Scopus, and 72 in Web of Science. Although the search window spans January 2015 to October 2025, the earliest included study dates to van Mulken et al. (2020), as dedicated microsurgical platforms only entered first‐in‐human use around that time; earlier records were largely preclinical/ex vivo work or general‐purpose‐platform studies using the robot only for flap harvest rather than the anastomosis itself, and were excluded accordingly. One general‐purpose‐platform study (Chang et al. 2020, da Vinci Xi) was retained because it met the intervention criterion directly, a robot‐performed nerve graft coaptation, confirming eligibility depended on the procedure performed, not the platform brand. Title and abstract screening, followed by full‐text review, were performed independently by two reviewers; discrepancies were resolved by consensus or third‐reviewer consultation. Following screening, 21 full‐text articles were selected for final synthesis.

Inclusion criteria: original research (RCTs, prospective/retrospective cohort studies, or case series with > 5 patients) reporting human clinical experience, published in English or German. Exclusion criteria: animal/preclinical studies, ex vivo training assessments, comparative reviews, and conference abstracts without full‐text data.

### 2.2 Data Extraction and Quality Assessment

Data were extracted independently by two reviewers using a standardized template capturing author/year, platform, design, indication, sample size, flap survival, anastomosis patency, complications, learning‐curve metrics, and ergonomic/technology findings.

Study quality was assessed using established tools: Cochrane RoB‐2 for the sole RCT (van Mulken et al. 2020), ROBINS‐I for non‐randomized interventional studies, and MINORS for case series. Full item‐by‐item scoring with rationale for all 21 studies is provided in Table S1.

### 2.3 Data Synthesis

Given substantial heterogeneity in surgical indication, platform, and methodology, formal meta‐analysis was not appropriate; data were collated as a Narrative Synthesis across clinical outcomes, safety, learning‐curve progression, and technological impact.

## 3 Results

**FIGURE 1:**
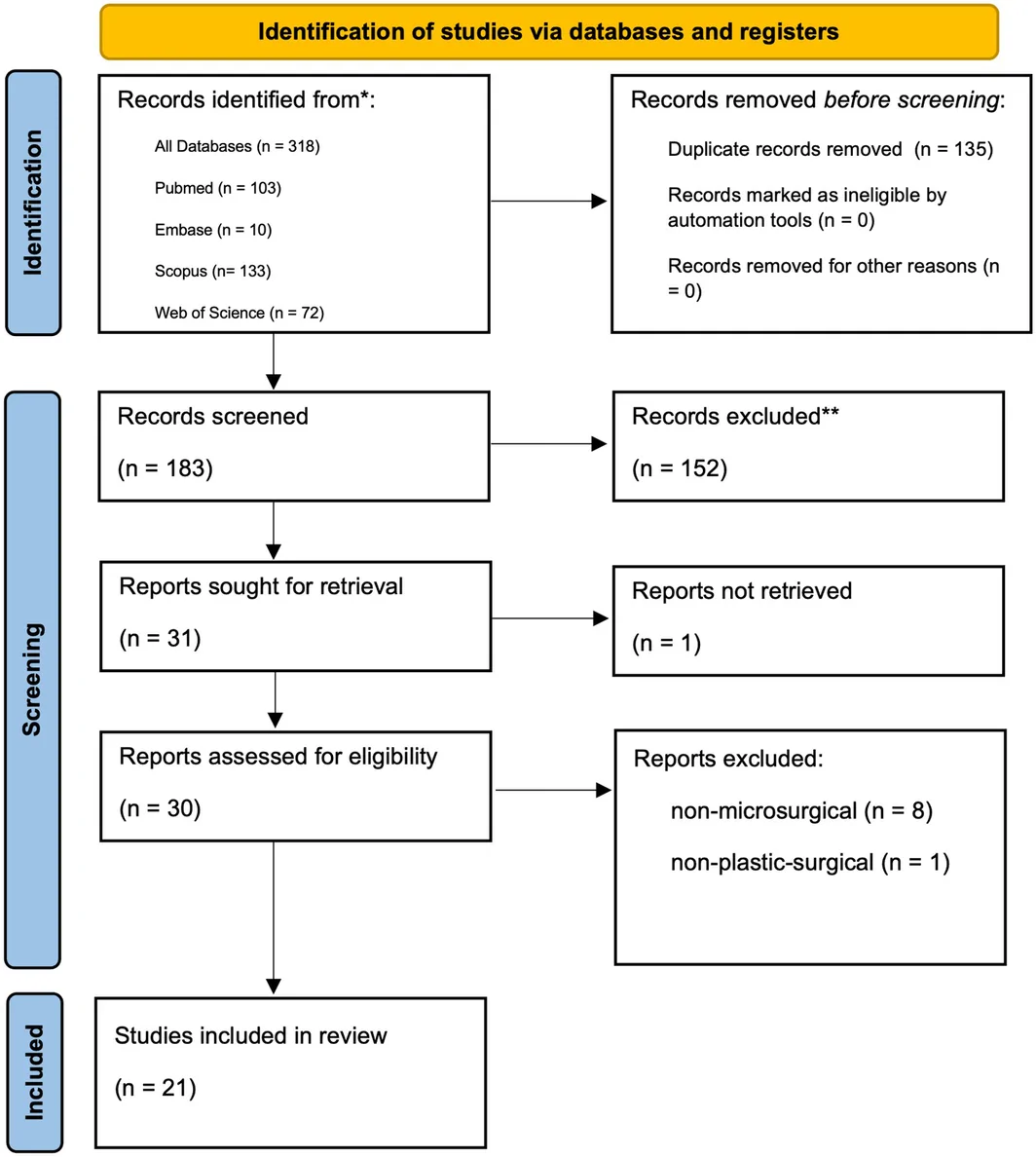
PRISMA 2020 flow diagram for new systematic reviews which included searches of databases and registers only. *Source:* Page MJ, et al. BMJ 2021;372:n71. doi:10.1136/bmj.n71. This work is licensed under CC BY 4.0. Records identified: PubMed/MEDLINE *n* = 103, Embase *n* = 10, Scopus *n* = 133, Web of Science *n* = 72 (total *n* = 318).

The search and screening process (Figure 1) yielded 318 records: PubMed/MEDLINE (*n* = 103), Embase (*n* = 10), Scopus (*n* = 133), and Web of Science (*n* = 72). After removing 135 duplicates, 183 records were screened; 152 were excluded at title/abstract level, leaving 31 reports sought for retrieval (1 not retrieved) and 30 assessed in full text. Nine were excluded at full‐text stage (8 non‐microsurgical, 1 non‐plastic‐surgical), leaving 21 studies for synthesis.

Baseline characteristics, study designs, interventions, and methodological quality for all 21 included studies are summarized in Table 1; the cohort predominantly evaluated the Symani and MUSA platforms across reconstructive indications.

**TABLE 1:** Study characteristics and risk of bias assessment for the 21 included studies, by robotic platform (Symani, MUSA, da Vinci), study design, and indication; quality assessed using MINORS, ROBINS‐I, and RoB‐2.

ID	Author/year	Platform	Design	Intervention	*N*	RoB assessment	Primary risk domain
1	Watson et al. (2025)	Symani	Retrospective case series	Free flap (scalp)	6	MINORS (high risk)	Lack of comparator
2	Konneker et al. (2025)	Symani	Retrospective feasibility study	Free tissue transfer (extremities)	8	MINORS (high risk)	Lack of comparator
3	Mori et al. (2024)	Symani	Retrospective case series	Free flap reconstruction	16	MINORS (high risk)	Lack of comparator
4	Weinzierl et al. (2023)	Symani	Retrospective case series	VLNT/LVA	8	MINORS (high risk)	Lack of comparator
5	von Reibnitz et al. (2024)	Symani	Retrospective case series	Lymphatic tissue transfer/LVA	67	MINORS (high risk)	Lack of comparator
6	Struebing, Bigdeli, Weigel, et al. (2024)	Symani	Prospective case series	Free flap, TMR, LVA	50	MINORS (high risk)	Lack of comparator
7	Barbon et al. (2022)	Symani	Retrospective case series	LVA, free flaps, nerve coaptation	22	MINORS (high risk)	Lack of comparator
8	Struebing, Boecker, et al. (2024)	Symani	Prospective case series	Free flap, nerve surgery, LVA	100	MINORS (high risk)	Lack of comparator
9	Sorensen et al. (2025)	Symani	Consecutive case series	Micro‐/super‐microsurgery/LVA	12	MINORS (high risk)	Lack of comparator
10	Dastagir et al. (2024)	Symani	Retrospective cohort	Microvascular anastomoses (trauma)	21 (10R/11M)	ROBINS‐I (serious)	Bias due to confounding
11	Gorji et al. (2024)	Symani/RoboticScope	Retrospective case series	Free flap reconstruction	23	MINORS (high risk)	Lack of comparator
12	Lindenblatt et al. (2022)	Symani	Retrospective case series	Lymphatic surgery (LVA, LLNA, VLNT)	5	MINORS (high risk)	Lack of comparator
13	Aman et al. (2024)	Symani	Prospective case series	Peripheral nerve surgery	19	MINORS (high risk)	Lack of comparator
14	Chen et al. (2025)	Symani	Retrospective case series	LNVA	22	MINORS (high risk)	Lack of comparator
15	Struebing et al. (2025)	Symani	Prospective observational study	RAMS across various applications	85	MINORS (high risk)	Lack of comparator
16	Struebing, Bigdeli, Boecker, et al. (2024)^a^	Symani	Prospective w/historical control	Free flap reconstruction (upper extremity)	16	ROBINS‐I (critical)	Bias due to confounding (historical control)
17	Chang et al. (2020)	da Vinci Xi	Retrospective case series	Sympathetic nerve reconstruction	7	MINORS (high risk)	Lack of comparator
18	van Mulken et al. (2020)	MUSA	Randomized pilot trial	LVA	20 (8R/12M)	RoB‐2 (high)	Performance bias; detection bias; deviation from intervention
19	Reilly et al. (2024)	MUSA‐2	Prospective implementation study	LVA	12	MINORS (high risk)	Lack of comparator
20	Debeij et al. (2024)	MUSA	Combined data (RCT + Pilot)	LVA, nerve repair, free tissue	35 procedures	MINORS (Mod)/RoB‐2 (high)	Deviation bias; statistical/selection bias
21	Beier et al. (2023)^a^	Symani	Prospective observational case series	Free flap reconstruction (arterial anast.)	23	MINORS (high risk)	Lack of comparator

Abbreviations: LNVA, lymph node‐to‐vein anastomosis; LVA, lymphaticovenous anastomosis; MINORS, methodological index for non‐randomized studies; RAMS, robot‐assisted microsurgery; RCT, randomized controlled trial; RoB‐2, revised cochrane risk‐of‐bias tool for randomized trials; ROBINS‐I, risk of bias in non‐randomized studies of interventions; TMR, Targeted Muscle Reinnervation; VLNT, vascularized lymph node transfer.

^a^ Struebing et al. (2025) (ID 8, 100‐case series) includes the 50‐case cohort published as Struebing, Bigdeli, Boecker, et al. (2024) (ID 6); overlapping cases are footnoted in Table 2 to avoid duplicate counting in the narrative synthesis.

### 3.1 Clinical Outcomes: Free Flap and Supermicrosurgery Efficacy

#### 3.1.1 Free Flap Reconstruction (Symani)

High‐volume series (Struebing, Bigdeli, Weigel, et al. 2024; Beier et al. 2023) reported aggregate flap survival rates of 96%–100% (Table 2), non‐inferior to conventional manual microsurgery benchmarks. Beier et al. (2023) reported 22/23 flap survivals (95.7%), with most complications successfully managed robotically. The Struebing et al. series represents an evolving cohort from a single center: the 100‐case experience (Struebing, Boecker, Vollbach, et al. 2024) includes the initial 50‐case series (Struebing, Bigdeli, Weigel, et al. 2024); data extraction avoided redundant patient counting in the synthesis.

**TABLE 2:** Aggregated clinical and safety outcomes of RAMS studies.

ID	Author/year	Platform	Indication	*N* (procedures/anast.)	Flap survival (%)	Anastomosis patency (%)	Major complication (%)
1	Watson et al. (2025)	Symani	Free flap reconstruction	6	100%	100%	0%
2	Konneker et al. (2025)	Symani	Free tissue transfer	8	100%	100%	0%
3	Mori et al. (2024)	Symani	Free flap reconstruction	16 (40 anast.)	Approx. 94% (1 partial necrosis)	100%	0%
4	Weinzierl et al. (2023)	Symani	VLNT/LVA	8	100% (omental flaps)	100%	0%
5	von Reibnitz et al. (2024)	Symani	LVA/LTT	67 (100 anast.)	N/A (flaps buried)	100%	7.5% (infection, hematoma)
6^a^	Struebing, Bigdeli, Weigel, et al. (2024)	Symani	Free flap, nerve, LVA	50 (94 anast.)	98% (no complete loss)	Approx. 97% (3 microvasc. compromise)	12%
7	Barbon et al. (2022)	Symani	LVA, arterial, nerve coaptation	22 (32 anast.)	N/A	97.5%	4.5% (1 thrombosis)
8^a^	Struebing et al. (2025)	Symani	Free flap, nerve, LVA	100 (159 anast.)	97.3% (2 complete losses)	96.6% (3 conversions)	12%
9	Sorensen et al. (2025)	Symani	Free flap/LVA	12	N/A	High (qualitative)	N/A
10	Dastagir et al. (2024)	Symani	Microvascular anast. (trauma)	21 (31 anast. rob.)	N/A	100%	0%
11	Gorji et al. (2024)	Symani/RoboticScope	Free flap reconstruction	23	95.7% (1 total loss)	Approx. 96% (1 failed)	8.7% (revision of anastomosis)
12	Lindenblatt et al. (2022)	Symani	Lymphatic surgery	5 (10 anast.)	N/A	100%	0%
13	Aman et al. (2024)	Symani	Peripheral nerve surgery	19 (25 coapt.)	N/A	N/A	5% (1 flap loss)
14	Chen et al. (2025)	Symani	LNVA	22	N/A	100%	0%
15	Struebing et al. (2025)	Symani	RAMS applications (workflow)	85	N/A	N/A	N/A
16	Struebing, Boecker, et al. (2024)	Symani	Free flap reconstruction (upper ext.)	16	100%	100%	6.25% (1 arterial thrombosis)
17	Chang et al. (2020)	da Vinci Xi	Sympathetic nerve reconstruction	7	N/A	N/A	N/A
18	van Mulken et al. (2020)	MUSA	LVA (RCT pilot)	8 (robotic group)	N/A	100%	0%
19	Reilly et al. (2024)	MUSA‐2	LVA	12	N/A	100%	0%
20	Debeij et al. (2024)	MUSA	LVA, nerve, free tissue	35 procedures	N/A	100% (intraop)	0%
21	Beier et al. (2023)	Symani	Free flap reconstruction	23	95.7% (1 flap loss)	High (not quantified)	4.3% (1 flap loss)

*Note:* Major complication rate refers to complete flap loss, major thrombosis requiring revision, or severe non‐flap‐related events.

Abbreviations: M, manual group; N/A, not assessed/applicable; R, robotic group.

^a^ Rows 6 and 8 (Struebing, Bigdeli, Boecker, et al. 2024; Struebing et al. 2025) represent overlapping cohorts from the same center: the 100‐case series (ID 8) includes the 50 cases reported in ID 6. Both are shown for completeness, but the 50 overlapping cases are counted once in any aggregate totals derived from this table.

#### 3.1.2 Supermicrosurgery (MUSA and Symani)

In lymphatic surgery, particularly LVA, RAMS facilitated high precision. Studies using MUSA (van Mulken et al. 2020) and Symani (von Reibnitz et al. 2024) uniformly reported 100% anastomosis patency, confirmed by intraoperative ICG lymphangiography, enabling consistent 10–0 to 12–0 suture placement in the smallest structures and deep anatomical planes (Weinzierl et al. 2023).

#### 3.1.3 Conversion and Failure Rates

Across studies reporting this metric, conversion or outright technical failure was infrequent: Struebing, Boecker, et al. (2024) reported conversion in 3/100 patients (3%); Gorji et al. (2024) reported conversion in 1/23 cases (4.3%), plus an 8.7% rate of arterial anastomosis revision; von Reibnitz et al. (2024) reported 7.5% wound infection/hematoma unrelated to device failure. No study reported an intraoperative robotic malfunction requiring emergency conversion. Reporting of this endpoint was nonetheless inconsistent across studies, limiting pooled estimation (see Limitations).

### 3.2 Learning Curve Dynamics

Studies reporting time‐based metrics consistently demonstrated a steep learning curve for anastomotic procedures in the initial clinical phase.

#### 3.2.1 Quantitative Improvement in Anastomosis Duration

Mean robot‐assisted anastomosis duration significantly decreased with increased surgical experience across all systems and procedures (reductions of 51%–66% across studies). The greatest reduction occurred in arterial anastomosis during omental flap transfer to the deep axilla, from 59 min (1st patient) to 20.0 ± 2.8 min (subsequent patients) (Weinzierl et al. 2023), a 66.1% reduction, supporting the hypothesis that robotic assistance is especially advantageous in deep anatomical regions. Similar steep declines occurred in other supermicrosurgical procedures: for LNVA (Symani), duration fell 51.4% from the 1st case (37.0 min) to the 22nd (18.0 min) (Chen et al. 2025); for LVA (MUSA), a comparable 51.5% reduction occurred from 33.0 min to 16.0 min (van Mulken et al. 2020).

#### 3.2.2 Plateauing and Comparison to Conventional Technique

**FIGURE 2:**
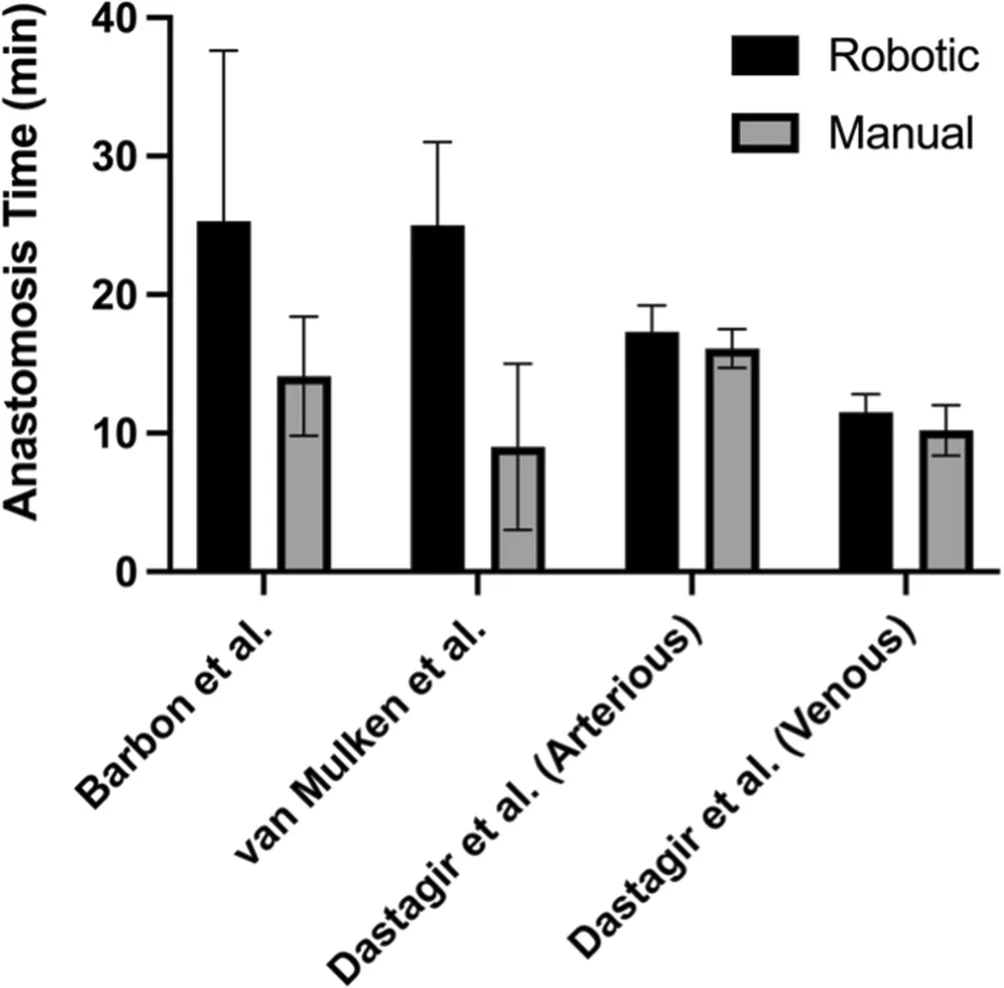
Comparison of mean anastomosis times for RAMS vs. manual techniques across four study cohorts: Barbon et al. (2022, mixed microsurgical procedures including LVA, LLA, arterial, and nerve anastomoses), van Mulken et al. (2020, randomized pilot trial for LVA), and Dastagir et al. (2024, arterial and venous anastomoses < 0.8 mm in trauma care, reported separately). Data presented as mean ± SD.

**FIGURE 3:**
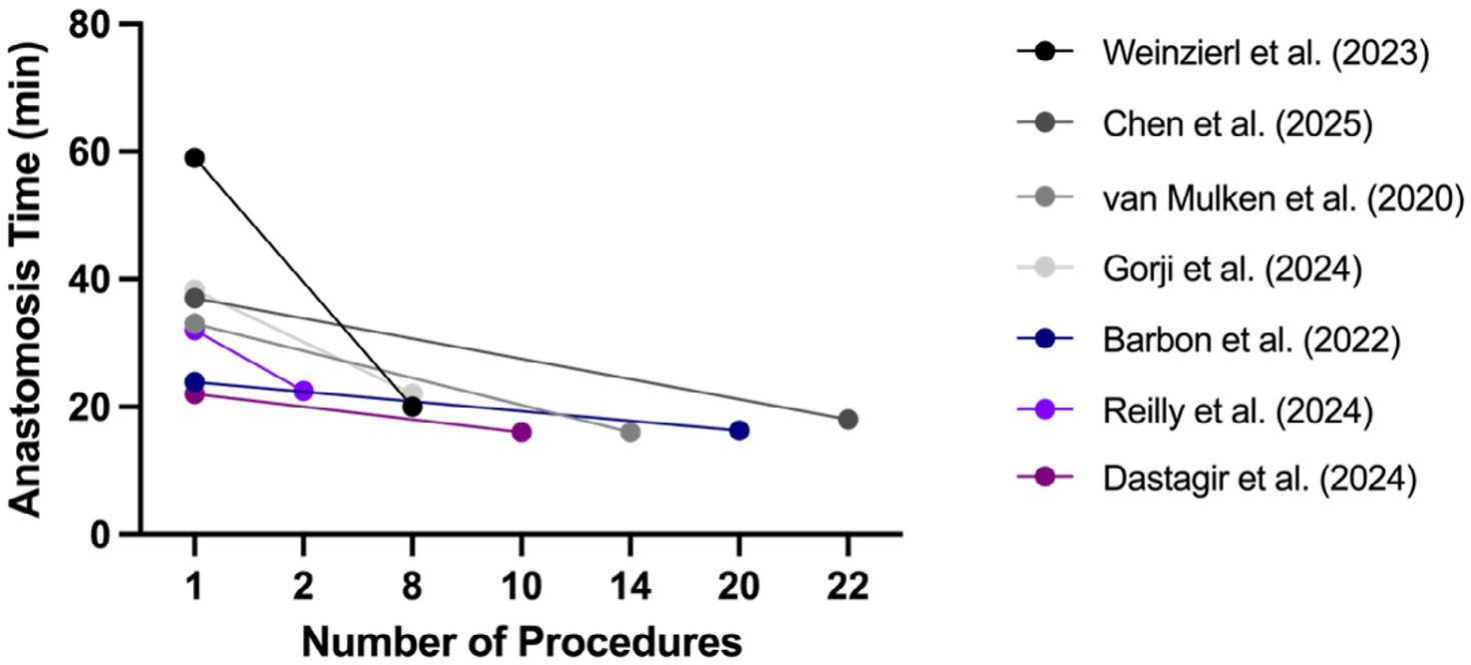
Learning curve progression in RAMS. XY‐plot correlating mean anastomosis time (min) with cumulative case number. Connecting lines visualize the trajectory of surgical efficiency from the initial case(s) to study conclusion; a steeper negative slope indicates more rapid technical acquisition. Data points represent study‐specific milestones (e.g., 1st case vs. 22nd case).

Following this initial improvement, robot‐assisted anastomosis times begin to approach the conventional manual benchmark (approx. 14–16 min) (Figure 2). In LVA cases (Symani), mean duration dropped from 23.9 ± 6.8 min (first cohort) to 16.3 ± 6.1 min (second cohort) (Barbon et al. 2022) (Figure 3). However, a large nerve‐coaptation series found no significant correlation between case number and time per stitch in later phases (Aman et al. 2024), suggesting time reduction occurs early before the curve plateaus.

##### 3.2.2.1 Manual Comparator Benchmarks

Where studies included a direct manual comparator, robotic times remained longer even after the learning phase, though the gap narrowed with experience. Barbon et al. (2022): 14.1 ± 4.3 min manual vs. 25.3 ± 12.3 min robotic (mixed procedures, *p* < 0.01). Dastagir et al. (2024): no significant difference for arterial (17.3 ± 1.9 vs. 16.1 ± 1.4 min) or venous (11.5 ± 1.3 vs. 10.2 ± 1.8 min) anastomoses in a trauma cohort. van Mulken et al. (2020): 9 ± 6 min manual vs. 25 ± 6 min robotic LVA. Debeij et al. (2024) cited historical/literature benchmarks without a prospective comparator. These figures, from the four studies with an explicit manual control group, give a consolidated reference point beyond the robotic‐arm data in Figures 2 and 3.

### 3.3 Technological Advancements and Trends

The evolution of dedicated RAMS platforms from 2020 to 2025 reveals distinct technological trends (Table 3) across two main system categories.

**TABLE 3:** Comparative technological specifications of RAMS platforms: Symani, MUSA, and da Vinci Xi, comparing motion scaling, degrees of freedom (DoF), and instrument design, and highlighting the universal absence of integrated haptic feedback.

Platform	Motion scaling	Tremor filtration	DoF	Instrument type	Haptic feedback	Visualization
Symani	7×–20×	Yes	7 DoF	Wristed Micro‐Instruments	No	Microscope & 3D Exoscope
MUSA	5×–20×	Yes	Limited (table‐mounted)	Standard Micro‐Instruments	No	Standard Microscope
da Vinci	3×–10×	Yes	7 DoF	Wristed Endoscopic Instruments	No	Endoscopic 3D (not microscope‐compatible)

#### 3.3.1 System Evolution and Application Scope

The earliest dedicated platform, MUSA (van Mulken et al. 2020), focused primarily on highly specialized supermicrosurgery (LVA) involving vessels < 0.8 mm, with initial implementation centered on feasibility and learning curve for these ultra‐delicate procedures.

The later Symani Surgical System (first‐in‐human use 2022, high‐volume series 2024/2025) offers a broader application scope, maintaining supermicrosurgical capabilities (motion scaling to 20:1, tremor filtration) while enabling high‐volume free flap reconstruction (Beier et al. 2023) and peripheral nerve surgery (Aman et al. 2024) via fully wristed 7‐DoF micro‐instruments with greater dexterity than earlier systems.

#### 3.3.2 Visualization Technology Shift

A notable trend is the shift from the traditional optical microscope toward digital, telemetric visualization. Early Symani studies (Lindenblatt et al. 2022) used the optical microscope alongside the robot, while more recent publications (Struebing, Boecker, Vollbach, et al. 2024; Gorji et al. 2024) show increasing integration of 4 K 3D exoscopes or fully telemetric systems, driven by improved surgeon ergonomics and an immersive stereoscopic view for the whole team. Gorji et al. (2024) reported the most advanced strategy, a fully telemetric setup.

#### 3.3.3 Haptic Feedback Limitation

**FIGURE 4:**
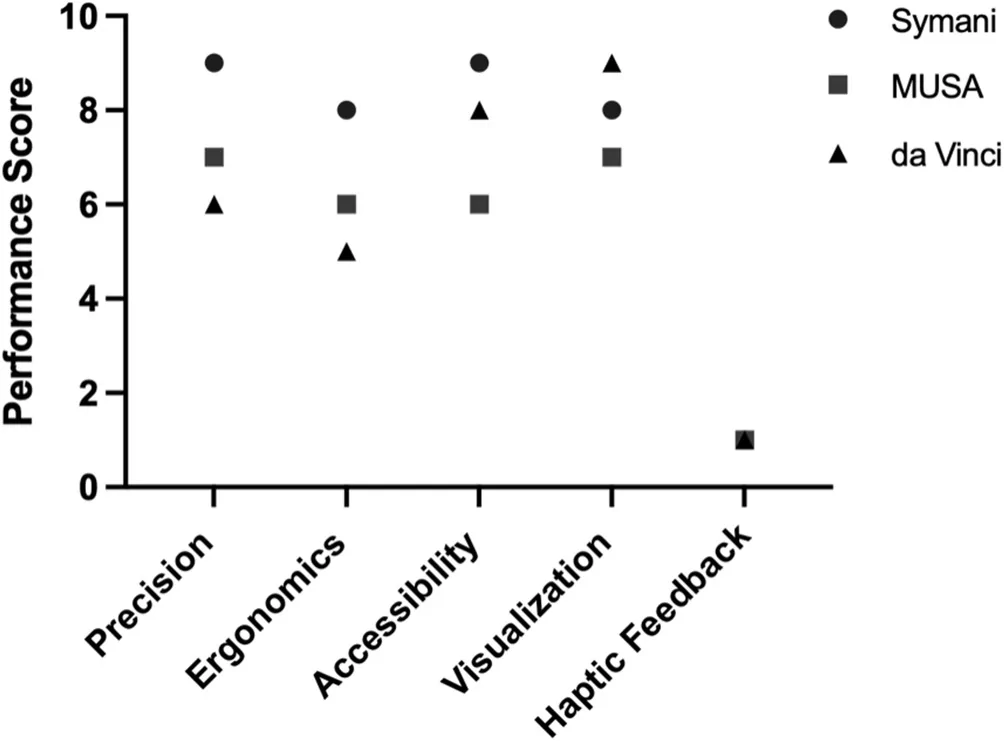
Comparative technological evaluation of RAMS platforms. Grouped scatter plot displaying performance scores (0–10) for the Symani Surgical System, MUSA, and da Vinci Xi across five technological domains: Precision (motion scaling and tremor filtration), Ergonomics (surgeon posture and fatigue reduction), Accessibility (instrument dexterity and degrees of freedom), Visualization (3D and exoscope compatibility), and Haptic Feedback (mechanical sensitivity and tissue feel). Scores represent the authors' qualitative synthesis of technical specifications and reported clinical experience, not data extracted directly or quantitatively from the included studies.

Despite advances in dexterity and visualization, the fundamental limitation across all dedicated platforms (MUSA and Symani) remains the universal absence of integrated haptic feedback (Figure 4), forcing surgeons to rely on visual‐force estimation, or “see‐feel,” cited as the primary technical drawback and a major element of the learning curve (Lindenblatt et al. 2022).

## 4 Discussion

**FIGURE 5:**
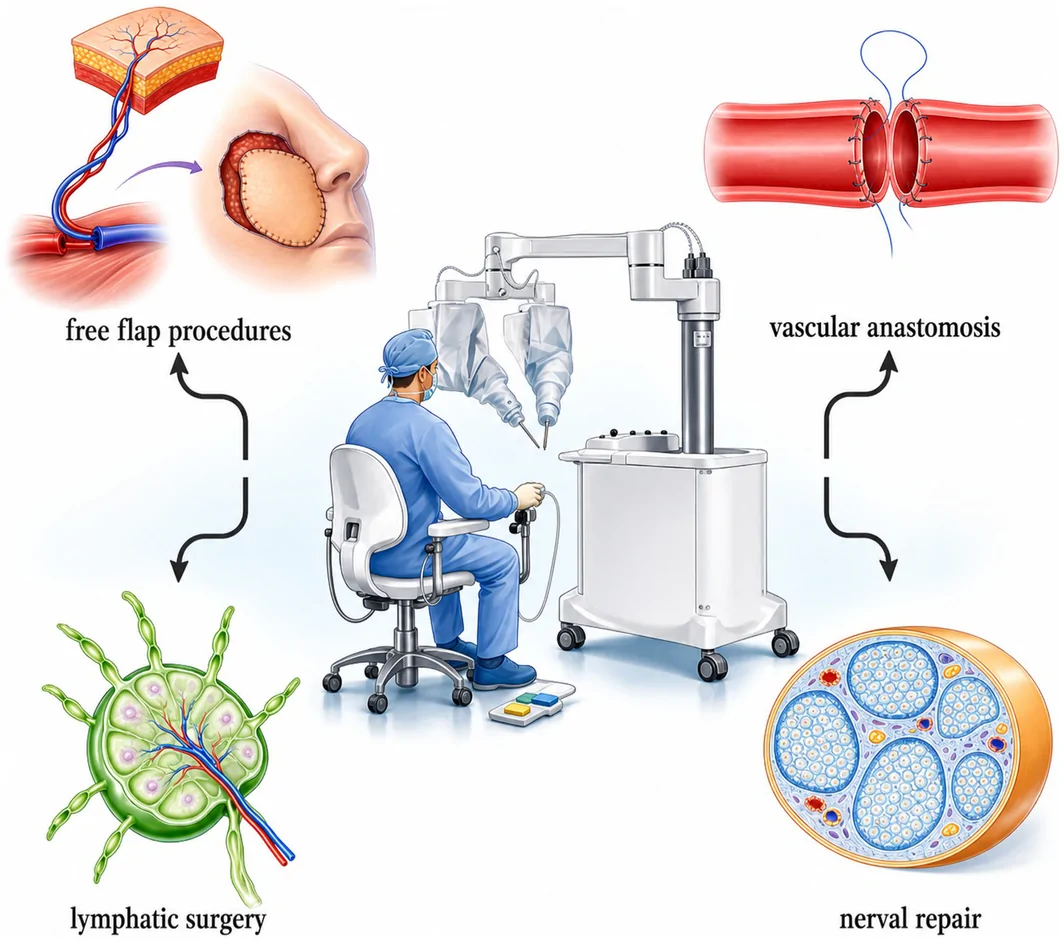
Clinical Application Domains and Reconstructive Indications of Dedicated RAMS Platforms. Illustration summarizing the primary surgical fields where robotic‐assisted microsurgery has established clinical utility: Free flap procedures, peripheral nerve repair, lymphatic supermicrosurgery (LVA, VLNT), and standardized vascular anastomosis.

This review confirms that dedicated RAMS platforms have transitioned from pre‐clinical feasibility to safe, effective clinical tools, with the most compelling evidence in two areas: high‐volume free flap reconstruction and delicate supermicrosurgery (Figure 5).

Compared with recent, broader reviews of robotic‐assisted plastic and reconstructive surgery (Ruccia et al. 2024; Kawashima et al. 2025), which span heterogeneous applications including breast, craniofacial, and aesthetic procedures, this review's exclusive focus on dedicated microsurgical platforms allowed a more granular, procedure‐specific synthesis. Unlike a supermicrosurgery‐focused review restricted to vessels < 0.8 mm without formal risk‐of‐bias appraisal (Brown et al. 2025), this review incorporated free flap reconstruction and nerve repair alongside supermicrosurgery, applied structured risk‐of‐bias assessment (RoB‐2, ROBINS‐I, MINORS) to all 21 studies, and consolidated learning‐curve and manual‐comparator time data quantitatively (Table 2, Figures 2 and 3) rather than narratively, providing a more actionable evidence base for centers weighing RAMS adoption.

Reported aggregate flap survival rates (96%–100%) align with or surpass manual‐microsurgery benchmarks, consistent with meticulous vessel preparation and tension‐free anastomosis being fundamental to flap survival (Nikkhah et al. 2023); robotic assistance appears to maintain and potentially enhance these standards through added stability during these critical steps. This is reassuring for concerns that added system complexity might compromise patient safety during adoption, though this should be interpreted cautiously given the evidence base is dominated by non‐randomized, single‐center series from high‐volume, early‐adopting centers. The intrinsic advantages of tremor filtration and high‐fidelity motion scaling instead appear to translate into reliable, high‐quality anastomoses, even with the small‐caliber vessels of perforator‐to‐perforator or supermicrosurgical settings.

The uniform LVA patency (100%) highlights the true niche of these robots: procedures where human physiological limits are routinely approached. Achieving patency below 0.8 mm represents the technical frontier of reconstruction, requiring precise 11–0/12–0 suture handling under extreme magnification (Nikkhah et al. 2023), and RAMS may lower this technical barrier through motion scaling and tremor filtration. However, despite identical patency rates, the van Mulken RCT's qualitative assessment initially favored the manual group: anastomotic quality scores (SAMS, UWOMSA) were significantly higher manually (e.g., SAMS 4.0 vs. 3.2), underlining that clinical success can be achieved even while “microsurgical elegance” and suture symmetry are initially affected by the lack of haptic feedback and the “see‐feel” transition.

One‐year follow‐up data from the first RCT in robotic LVA (van Mulken et al. 2022) provide critical evidence of long‐term durability (van Mulken et al. 2022): the MUSA robot's initial technical precision translated into sustained clinical improvements in breast cancer‐related lymphedema, with significant limb‐volume reduction and quality‐of‐life gains comparable to the manual gold standard at 12 months, confirming that tremor filtration and motion scaling yield reliable long‐term therapeutic outcomes, not merely intraoperative advantages.

A central theme is the trade‐off between precision and initial efficiency: prolonged anastomosis times carry a tangible procedural cost, for example, a mean of 69 min (range 31–125 min) for arterial end‐to‐end anastomoses in Beier et al.'s series (Beier et al. 2023), or a single first case of 59 min falling to a mean of 20 min in Weinzierl et al.'s series (Section 3.2.1) (Weinzierl et al. 2023), underscoring the need for dedicated training. Crucially, the steep learning curve suggests this time penalty is transient and overcome with case volume, a positive sign that centers can reach a proficiency plateau relatively quickly. For surgeons, the training‐time investment is balanced by ergonomic benefits from the teleoperated console, which could extend career longevity and reduce fatigue.

The major technical challenge remains the absence of haptic feedback: substituting tactile sensation with a visual “see‐feel” judgment of tension is a learned skill all adopting surgeons must acquire. Its clinical impact should be contextualized within existing training paradigms, since microsurgeons already rely on visual cues to assess force, a reliance well documented in the microsurgical and robotic‐training literature (Cutler et al. 2013; Jourdes et al. 2022). In supermicrosurgery, structural limits of vessel or thread are often reached before tactile resistance would be perceptible, so haptic feedback here may be counterproductive; the required “see‐feel” proficiency represents a refined extension of an existing skill set rather than an entirely new burden.

This remains a key area for future innovation: high‐fidelity haptic technology could further flatten the learning curve, improving speed and safety during adoption.

The trend toward digital 3D visualization marks an important ergonomic and collaborative advance, though visualization‐system choice requires careful institutional consideration given implications for setup, ergonomics, and cost. A notable limitation of exoscopic visualization is restricted depth‐of‐field; unlike endoscopic systems (e.g., da Vinci) that access recessed areas directly (e.g., posterior oropharynx in transoral surgery), exoscopes need an unobstructed external line‐of‐sight, difficult to maintain in deep or narrow corridors.

This review's findings must be interpreted in light of its limitations. The evidence base consists mainly of non‐randomized, single‐center case series; while these offer valuable real‐world insight into clinical use and learning‐curve dynamics, they lack the external validity of large‐scale RCTs. The most robust evidence, the pilot LVA RCT, supports the technical advantages, but more RCTs comparing long‐term functional and patient‐reported outcomes across applications are needed.

The current literature may be subject to publication bias favoring positive early outcomes. Heterogeneity across procedures and surgeon experience prevented formal meta‐analysis, and conversion/failure reporting was inconsistent. Long‐term functional outcomes remain scarce beyond one‐year lymphatic‐surgery follow‐up, particularly for nerve reconstructions. Three further limitations warrant note: the restriction to English‐ and German‐language publications may have introduced language bias; formal certainty‐of‐evidence grading (e.g., GRADE) was not undertaken, as the near‐entirely non‐randomized evidence base would default to very‐low certainty, so we relied instead on study‐level risk‐of‐bias assessment (Table 1, Table S1); and we did not statistically correlate technical parameters (e.g., motion‐scaling ratio, DoF) with outcomes, as heterogeneity and small series size precluded meaningful quantitative analysis, an important direction for future research.

A further barrier to adoption is the lack of rigorous cost‐effectiveness analyses, which would need to account for purchase price, maintenance contracts, instrument costs, compatible visualization systems, and surgeon/theater‐team time during the learning‐curve phase. As none of the included studies reported formal cost data, these estimates remain qualitative; future research must establish whether clinical and ergonomic benefits justify these investments relative to conventional microsurgery.

## 5 Conclusion

Dedicated RAMS platforms, such as Symani and MUSA, are safe and effective tools for complex micro‐ and supermicrosurgical reconstruction, with clinical outcomes (flap survival, anastomosis patency) non‐inferior to conventional manual techniques. Their core advantages, tremor filtration and high‐ratio motion scaling, are particularly beneficial in supermicrosurgery and deep, confined anatomical planes. While a significant time penalty is initially associated with the learning curve, proficiency is gained rapidly, and technological evolution shows a clear trend toward broader application (Symani) and digital 3D exoscopic visualization for improved ergonomics and collaboration.

Future research should prioritize: (Debeij et al. 2024) integrated haptic feedback to further flatten the learning curve; (Nikkhah et al. 2023) large‐scale, multi‐center RCTs on long‐term functional outcomes, particularly in nerve repair, where evidence is most limited; (Lee et al. 2012) standardized, validated patient‐reported outcome measures across platforms; (Tan et al. 2018) consistent, prospectively defined conversion/failure‐rate reporting; and (von Reibnitz et al. 2024) formal cost‐effectiveness analyses establishing long‐term clinical and economic value versus the manual gold standard.

## Author Contributions

S.M.K. and L.A. conceived and designed the study. S.M.K. conducted the literature search and led study screening, data extraction, risk‐of‐bias assessment, and manuscript drafting. M.E. independently performed study screening, full‐text review, and data extraction, and critically revised the manuscript. L.A. critically revised the manuscript for important intellectual content. D.M.K., P.B., and D.N. supervised the review, provided critical intellectual input on study design and data interpretation, and critically revised the manuscript for important intellectual content. All authors read and approved the final version of the manuscript for submission.

## Funding

The authors have nothing to report.

## Ethics Statement

Ethical approval was not required for this study, as it is a systematic review based entirely on previously published, publicly available peer‐reviewed literature and does not involve direct intervention with human participants or animals.

## Conflicts of Interest

The authors declare no conflicts of interest.

## Supporting information


**Table S1:** Detailed risk‐of‐bias assessment for all 21 included studies.

## Data Availability

This is a systematic review; no new primary data were generated. All data underlying the findings reported are derived from previously published, publicly available studies, which are cited within the manuscript and listed in the reference list. The data extraction spreadsheets and risk‐of‐bias assessment tables compiled during this review are available from the corresponding author upon reasonable request.
