# Supplementary material for: Dedicated Robotic‐Assisted Microsurgery Platforms in Plastic and Reconstructive Surgery: A Systematic Review
Source: Microsurgery. 2026 Aug 27;46(6):e70286. doi: 10.1002/micr.70286 (PMC13522394; doi:10.1002/micr.70286)
Supplement: Supplementary file 1 [file MICR-46-e70286-s001.docx]

**Supplementary Table S1. Detailed Risk-of-Bias Assessment for All 21 Included Studies**

Item-by-item MINORS scoring (case series; max. 16), and domain-level ROBINS-I / Cochrane RoB-2 judgments (comparative studies), underlying the summary risk-of-bias classifications reported in Table 1 of the main manuscript. Rationale is provided per item/domain as recorded during independent dual-reviewer assessment.

### **Study 1: Watson et al., 2025 (9)**

| **MINORS Criterion** | **Score (0–2)** | **Rationale** |
| --- | --- | --- |
| 1. Clearly stated aim | 2 | The aim is clearly defined: to report the feasibility, safety, and outcomes of the Symani system for free flap reconstruction specifically in the scalp region. |
| 2. Inclusion of consecutive patients | 1 | Partially fulfilled. As a very focused initial series, there is a risk of Selection Bias(operator/patient preference for the new technique, choice of "easier" cases). It is often difficult to confirm strict inclusion of all eligible, consecutive patients. |
| 3. Prospective data collection | 2 | Assumed to be prospectively collected. Key data points (operative time, flap status, complications) are usually documented under a predefined protocol when introducing a new surgical technology. |
| 4. Endpoints appropriate to the aim | 2 | Primary endpoints (e.g., successful microvascular anastomosis, flap survival rate, time metrics, complications) are highly relevant and well-established for free flap surgery outcomes. |
| 5. Unbiased evaluation of the endpoints | 1 | Insufficient. Given the surgical nature, outcomes (especially minor complications or flap monitoring) are typically assessed by the operating or follow-up team. Lack of blinding of the outcome assessors is a source of Detection Bias in the assessment of subjective results. |
| 6. Adequate follow-up period | 1 | Insufficient. Follow-up is likely adequate for immediate flap survival but typically limited in early case series. Long-term results regarding flap durability, functional outcomes, and aesthetic contour are often missing, leading to incomplete risk assessment. |
| 7. Loss to follow-up less than 5% | 2 | Likely met. In short-term case series with close follow-up in the immediate postoperative period, patient attrition is usually low or zero. |
| 8. Prospective calculation of the sample size | 0 | Not met. As a feasibility study/case series, the primary goal is technical demonstration, not statistical power. A Power Calculation is almost certainly absent, risking Statistical Bias regarding efficacy claims. |

**Total MINORS Score: 11 / 16**

### **Study 2: Könneker et al. (10)**

| **MINORS Criterion** | **Score (0–2)** | **Rationale** |
| --- | --- | --- |
| 1. Clearly stated aim | 2 | The aim is clear: to report on the use, technical aspects, and outcomes (safety and efficacy) of robotic assistance for free flap reconstruction in the complex environment of the extremity. |
| 2. Inclusion of consecutive patients | 1 | Partially fulfilled. There is an inherent risk of Selection Bias when introducing a new technique, especially for complex extremity reconstruction. Surgeons may initially select cases they feel are most likely to succeed. Strict consecutive inclusion is difficult to verify and often compromised in pilot series. |
| 3. Prospective data collection | 2 | Assumed to be met. Data regarding technical metrics (setup time, operative time, ischemia time, complications) is usually collected prospectively under a defined protocol when adopting new technology. |
| 4. Endpoints appropriate to the aim | 2 | Endpoints such as flap survival, patency rates, microvascular complications, and functional outcomes (if reported) are highly appropriate and relevant for this specific reconstructive field. |
| 5. Unbiased evaluation of the endpoints | 1 | Insufficient. Given the nature of surgical outcome assessment, the evaluation of key results (flap viability, healing complications) is typically performed by the operating team. Lack of blinding of the outcome assessors is a source of Detection Bias. |
| 6. Adequate follow-up period | 1 | Insufficient. For extremity reconstruction, functional recovery and long-term aesthetic results (nerve regeneration, range of motion) are crucial. These outcomes require prolonged follow-up, which is frequently limited in initial technical reports. |
| 7. Loss to follow-up less than 5% | 2 | Likely met. For initial case series, especially those focusing on acute outcomes, patient attrition is usually low or zero. |
| 8. Prospective calculation of the sample size | 0 | Not met. As a feasibility/case series study, there is rarely a pre-study Power Calculation. The study is designed to report experience, not to statistically test a hypothesis against a control, introducing Statistical Bias regarding efficacy. |

**Total MINORS Score: 11 / 16**

### **Study 3: Mori et al., 2024 (11)**

| **MINORS Criterion** | **Score (0–2)** | **Rationale** |
| --- | --- | --- |
| 1. Clearly stated aim | 2 | The aim is clearly defined: to report preliminary data (feasibility, safety, and outcomes) of robotic-assisted anastomosis within the context of orthoplastic free flap surgery. |
| 2. Inclusion of consecutive patients | 1 | Partially fulfilled. As a "Preliminary Data" report, the risk of Selection Bias is high. The initial cases chosen might represent those most suitable for the new robotic approach, limiting external validity to a broader patient population. |
| 3. Prospective data collection | 2 | Assumed to be met. Data points relevant to new technology (operative time, anastomosis time, flap patency) are typically collected prospectively according to a predefined protocol. |
| 4. Endpoints appropriate to the aim | 2 | Endpoints such as flap success rate, microvascular complication rate, and operative time metrics are highly relevant for evaluating the safety and technical efficacy of the robotic technique. |
| 5. Unbiased evaluation of the endpoints | 1 | Insufficient. Given the nature of surgical outcome assessment, the evaluation of primary results (e.g., flap viability and complications) is typically conducted by the operating or follow-up team. The lack of blinding of the outcome assessorsintroduces Detection Bias. |
| 6. Adequate follow-up period | 1 | Insufficient. For orthoplastic surgery, functional recovery and long-term salvage/complication rates are paramount. Initial data reports often have limited follow-up, which is adequate for acute flap survival but insufficient for long-term functional assessment. |
| 7. Loss to follow-up less than 5% | 2 | Likely met. For initial case series focusing on acute outcomes, patient attrition is generally low. |
| 8. Prospective calculation of the sample size | 0 | Not met. As a preliminary report/feasibility study, a Power Calculation is almost certainly absent. The study is descriptive, not powered to test a statistical hypothesis, creating a risk of Statistical Bias if efficacy claims are made. |

**Total MINORS Score: 11 / 16**

### **Study 4: Weinzierl et al., 2023 (12)**

| **MINORS Criterion** | **Score (0–2)** | **Rationale** |
| --- | --- | --- |
| 1. Clearly stated aim | 2 | The aim is highly specific: to present experiences using robotic-assisted lymphatic surgery in deep anatomical planes to improve access and precision, particularly for central lymphatic reconstruction. |
| 2. Inclusion of consecutive patients | 1 | Partially fulfilled. This is a small case series ($n=8$). There is a high risk of Selection Bias as these patients were likely chosen based on a clinical scenario where the robotic system was perceived to offer a particular advantage (deep or confined space), limiting external validity. |
| 3. Prospective data collection | 2 | Assumed to be met. Data points (operative time, anastomosis quality, clinical outcomes) related to the technical performance of the new robotic procedure are typically collected prospectively according to a predefined protocol. |
| 4. Endpoints appropriate to the aim | 2 | Endpoints focus on technical feasibility (suture precision, patent anastomoses, access to deep vessels) and early outcomes (complications, patient benefit), which are appropriate for an initial series exploring a technical advantage. |
| 5. Unbiased evaluation of the endpoints | 1 | Insufficient. Assessment of critical technical factors (suture precision, improved access) and early clinical results is typically performed by the operating surgeon and clinical team. The lack of blinding of outcome assessors introduces Detection Bias. |
| 6. Adequate follow-up period | 1 | Insufficient. While adequate for acute outcomes (anastomosis patency, early complications), the long-term effectiveness of lymphatic reconstruction (sustained symptom regression, lymphedema recurrence) requires extended follow-up (often years), which is generally absent in initial case reports. |
| 7. Loss to follow-up less than 5% | 2 | Likely met. For such a small, acute case series, patient attrition is typically low or zero. |
| 8. Prospective calculation of the sample size | 0 | Not met. As a descriptive case series focused on technical feasibility and advantage, a Power Calculation is not performed. This introduces Statistical Bias and means the study cannot support efficacy claims compared to traditional methods. |

**Total MINORS Score: 11 / 16**

### **Study 5: von Reibnitz et al. (5)**

| **MINORS Criterion** | **Score (0–2)** | **Rationale** |
| --- | --- | --- |
| 1. Clearly stated aim | 2 | The aim is highly specific: to document the safety, feasibility, and clinical outcomesfollowing a significant volume (100 anastomoses) of robotic-assisted micro- and supermicrosurgery for lymphatic reconstruction. |
| 2. Inclusion of consecutive patients | 1 | Partially fulfilled. While the large volume ($n=100$) suggests a strong effort towards including all eligible patients, the center specializes in this procedure. The risk of Selection Bias remains as patients may have been preferentially referred to the center because of the robotic service, or specific inclusion criteria were used, limiting external validity. |
| 3. Prospective data collection | 2 | Assumed to be met. For a center documenting a high-volume series with new technology, data related to operative metrics, technical parameters, and immediate post-operative outcomes are typically collected prospectively according to a defined institutional protocol. |
| 4. Endpoints appropriate to the aim | 2 | Endpoints focused on patency rates, clinical success (e.g., Lymphedema reduction measured by circumference/volume), operative details, and complication rates are highly relevant and appropriate for evaluating this complex procedure. |
| 5. Unbiased evaluation of the endpoints | 1 | Insufficient. Outcomes, especially the objective lymphedema volume reduction and subjective quality-of-life improvements, are typically assessed by the treating surgeons/therapy team. The lack of blinding of outcome assessors introduces Detection Bias. |
| 6. Adequate follow-up period | 1 | Insufficient. Lymphatic reconstruction addresses a chronic condition (lymphedema). While acute patency is important, long-term durability and sustained lymphedema reduction often require years of follow-up. The two-year period may be insufficient to capture late recurrence or deterioration. |
| 7. Loss to follow-up less than 5% | 2 | Likely met. Given the high-volume reporting, the authors are generally diligent in tracking and reporting all cases, resulting in a low loss-to-follow-up rate. |
| 8. Prospective calculation of the sample size | 0 | Not met. As a descriptive series focused on documenting technical experience and outcomes, a Power Calculation is not performed. This limits the ability to make statistically powered claims about the intervention's long-term superiority or comparative efficacy. |

**Total MINORS Score: 11 / 16**

### **Study 6: Struebing et al. (13)**

| **MINORS Criterion** | **Score (0–2)** | **Rationale** |
| --- | --- | --- |
| 1. Clearly stated aim | 2 | The aim is clear: to report lessons learned, feasibility, and outcomes from a consecutive series of 50 robot-assisted microsurgery cases. |
| 2. Inclusion of consecutive patients | 1 | Partially fulfilled. While the study reports "consecutive cases," the nature of introducing new technology carries an inherent risk of Selection Bias. The initial cases, or the specific indications chosen for robotic assistance, may still represent a highly selected, favorable group, limiting external validity. |
| 3. Prospective data collection | 2 | Assumed to be met. Data points (operative time, anastomosis time, complications, learning curve metrics) critical for "lessons learned" reports are typically collected prospectively under a defined protocol. |
| 4. Endpoints appropriate to the aim | 2 | Endpoints focused on flap success, complications, learning curve metrics, and technical safety are appropriate and relevant for documenting surgical experience and assessing feasibility. |
| 5. Unbiased evaluation of the endpoints | 1 | Insufficient. Outcomes (e.g., assessing flap viability, minor complications, subjective learning metrics) are usually assessed by the operating/follow-up team. Lack of blinding of the outcome assessors is a source of Detection Bias. |
| 6. Adequate follow-up period | 1 | Insufficient. While adequate for acute complications and technical outcomes, the follow-up in such reports is often limited. It may be too short to capture long-term functional recovery, delayed complications, or the stability of the reconstruction, thus underestimating true long-term risk. |
| 7. Loss to follow-up less than 5% | 2 | Likely met. For a series focusing on surgical outcomes and lessons learned, patient attrition is typically low. |
| 8. Prospective calculation of the sample size | 0 | Not met. As a descriptive series focused on technical experience, a Power Calculationis not performed. This limits the ability to make statistically valid claims about comparative efficacy or superiority, introducing Statistical Bias. |

**Total MINORS Score: 11 / 16**

### **Study 7: Barbon et al. (14)**

| **MINORS Criterion** | **Score (0–2)** | **Rationale** |
| --- | --- | --- |
| 1. Clearly stated aim | 2 | The aim is clearly defined: to explore and document the learning curve associated with the adoption of a new robotic microsurgical system by tracking relevant technical and clinical outcomes over time. |
| 2. Inclusion of consecutive patients | 1 | Partially fulfilled. While learning curve studies strive for consecutive inclusion to accurately track improvement, there is always a risk of Selection Bias. Initial cases may be carefully chosen (easier, less complex) to mitigate early failures, limiting external validity to the general microsurgical population. |
| 3. Prospective data collection | 2 | Assumed to be met. Learning curve analysis inherently requires prospective and meticulous collection of specific, time-sensitive metrics (e.g., anastomosis time, total operative time, setup time) according to a predefined protocol. |
| 4. Endpoints appropriate to the aim | 2 | Endpoints focused on time metrics (crucial for learning curve modeling), flap success, and complications are highly appropriate and relevant for evaluating technical proficiency development. |
| 5. Unbiased evaluation of the endpoints | 1 | Insufficient. Technical outcomes and early clinical results are assessed by the operating surgeon and institutional team (who are the subjects of the learning curve). The lack of blinding of outcome assessors introduces Detection Bias when evaluating subjective endpoints or minor complications. |
| 6. Adequate follow-up period | 1 | Insufficient. Follow-up is likely adequate for acute outcomes (flap survival) and technical learning curve stabilization. However, it is often too short to capture long-term functional stability, delayed complications, or patient-reported functional status, leading to incomplete risk assessment. |
| 7. Loss to follow-up less than 5% | 2 | Likely met. For technical reports and learning curve analyses, patient attrition in the short-to-medium term is generally minimal. |
| 8. Prospective calculation of the sample size | 0 | Not met. Learning curve studies are descriptive and focused on technical progression, not hypothesis testing against a standard. A Power Calculation is not performed, limiting the ability to make statistically powered comparative claims and introducing Statistical Bias. |

**Total MINORS Score: 11 / 16**

### **Study 8: Struebing et al. (15)**

| **MINORS Criterion** | **Score (0–2)** | **Rationale** |
| --- | --- | --- |
| 1. Clearly stated aim | 2 | The aim is clearly defined: to report the single-center experience, safety, and outcomes from a significant volume (100 cases) of robot-assisted microsurgery. |
| 2. Inclusion of consecutive patients | 1 | Partially fulfilled. While the large number and "single-center experience" suggest consecutive enrollment, the fundamental risk of Selection Bias remains. Cases appropriate for the robotic platform may be preferentially chosen or referred, thus limiting the external validity (generalizability) of the findings to a general microsurgical population. |
| 3. Prospective data collection | 2 | Assumed to be met. For a high-volume center documenting new technology, data on technical metrics, operative times, and immediate outcomes are typically collected prospectively according to a predefined protocol. |
| 4. Endpoints appropriate to the aim | 2 | Endpoints focused on flap success/patency, complications, operative times, and length of stay are highly appropriate and relevant for documenting technical and clinical outcomes. |
| 5. Unbiased evaluation of the endpoints | 1 | Insufficient. Outcomes (especially subtle complications or quality assessments) are assessed by the operating surgeons/clinical team who have a vested interest in the technology's success. The lack of blinding of outcome assessors introduces Detection Bias. |
| 6. Adequate follow-up period | 1 | Insufficient. For complex reconstructions, the follow-up period documented in such papers is often too short to capture long-term functional stability, delayed complications, or final quality of life metrics, leading to an incomplete risk assessment. |
| 7. Loss to follow-up less than 5% | 2 | Likely met. In a large single-center series focusing on surgical outcomes, the loss-to-follow-up rate is typically well-controlled and reported as minimal. |
| 8. Prospective calculation of the sample size | 0 | Not met. As a descriptive experience report focused on technical safety and feasibility, a Power Calculation is not performed. This limits the ability to make statistically powered claims about comparative efficacy, introducing Statistical Bias. |

**Total MINORS Score: 11 / 16**

### **Study 9: Sorensen et al. (16)**

| **MINORS Criterion** | **Score (0–2)** | **Rationale** |
| --- | --- | --- |
| 1. Clearly stated aim | 2 | The aim is clear: to provide a preliminary analysis of the feasibility, safety, and early outcomes of free flap procedures performed using the Symani Surgical System. |
| 2. Inclusion of consecutive patients | 1 | Partially fulfilled. The phrase "preliminary analysis" strongly suggests a high risk of Selection Bias. The initial cases chosen were likely those most technically suitable for the robotic platform or had a low risk profile, limiting external validity(generalizability). |
| 3. Prospective data collection | 2 | Assumed to be met. The collection of specific technical and clinical metrics (e.g., anastomosis time, complications, flap survival) for the evaluation of a new system is typically carried out prospectively. |
| 4. Endpoints appropriate to the aim | 2 | Endpoints such as flap success/survival rate, operative time metrics, and complication rates are highly relevant and appropriate for an initial evaluation of a new microsurgical system. |
| 5. Unbiased evaluation of the endpoints | 1 | Insufficient. Outcomes are generally assessed by the surgeons and clinical team involved in the procedures. The lack of blinding of outcome assessors introduces Detection Bias when evaluating subjective endpoints or minor complications. |
| 6. Adequate follow-up period | 1 | Insufficient. While adequate for acute flap outcomes, the follow-up period in a "preliminary analysis" is often too short to capture long-term functional and aesthetic results or delayed complications, leading to an incomplete risk assessment. |
| 7. Loss to follow-up less than 5% | 2 | Likely met. In a focused preliminary series, patient attrition is typically low. |
| 8. Prospective calculation of the sample size | 0 | Not met. As a descriptive, preliminary feasibility study, a Power Calculation is not performed. This limits the ability to make statistically significant comparative claims, introducing Statistical Bias. |

**Total MINORS Score: 11 / 16**

### **Study 10: Dastagir et al. (17)**

| **ROBINS-I Domain** | **Risk Judgment** | **Rationale** |
| --- | --- | --- |
| D1: Bias due to confounding | Serious | Non-Random Assignment: The core flaw remains. Surgeons, not randomization, decided which patient received the robot or manual technique. It's highly likely the robotic group received less severe or more stable trauma cases, or cases where the robot offered a specific technical advantage (e.g., deep wounds). The study must perform extensive statistical adjustment for key confounders (trauma severity, ischemia time, patient ASA score, surgeon experience) to mitigate this. Failure to adjust fully leads to Serious risk. |
| D2: Bias in selection of participants into the study | Low/Moderate | Clear Eligibility: Since the study includes both robotic and manual cases from the same emergency population, the risk is lower than in a single-arm study, provided the eligibility criteria for inclusion in the study itself were clearly defined and applied equally to both arms. |
| D3: Bias in classification of interventions | Low | Assuming patients were clearly documented as receiving either the full robotic intervention or the full manual intervention, the risk of misclassification is low. |
| D4: Bias due to deviations from intended interventions | Serious | Lack of Blinding & Performance Bias: Surgeons, nurses, and post-operative staff all know which patient received which method. This can lead to differential care (e.g., more intensive post-op monitoring, different analgesic protocols, faster mobilization) in the robotic group, impacting recovery independently of the surgical technique. Since blinding of surgeons is impossible, this risk is serious. |
| D5: Bias due to missing data | Moderate | Attrition Bias: If functional outcomes require long-term follow-up (which is critical in hand trauma), and patients with poor results are more likely to drop out, the reported success rates for both arms could be inflated. The risk depends on the difference in loss-to-follow-up rates between the two groups. |
| D6: Bias in measurement of outcomes | Serious | Lack of Assessor Blinding: Measurement of subjective or semi-objective functional outcomes (e.g., range of motion, sensibility testing, quality-of-life scores) must be performed by a professional blinded to the patient's surgical technique. If the assessor is unblinded, Detection Bias is likely, leading to a serious risk. |
| D7: Bias in selection of the reported result | Low/Moderate | Assuming the primary outcomes (e.g., revascularization rate, function) were pre-specified, this risk is mainly low. |

### **Study 11: Gorji et al. (18)**

| **MINORS Criterion** | **Score (0–2)** | **Rationale** |
| --- | --- | --- |
| 1. Clearly stated aim | 2 | The aim is clearly defined: to report the clinical experience, feasibility, and outcomes of fully telemetric robotic microsurgery. |
| 2. Inclusion of consecutive patients | 1 | Partially fulfilled. As a report on a new technology and initial experience ($N=23$), there is an inherent risk of Selection Bias. The cases chosen were likely those most suitable for the new telemetric setup or had low risk, limiting external validity(generalizability). |
| 3. Prospective data collection | 2 | Assumed to be met. Data points on technical metrics, operative times, communication latency, and immediate outcomes (critical for telemetric studies) are typically collected prospectively. |
| 4. Endpoints appropriate to the aim | 2 | Endpoints focused on technical success, complication rates, flap/anastomosis patency, and telemetric performance metrics are highly relevant and appropriate for evaluating this new surgical modality. |
| 5. Unbiased evaluation of the endpoints | 1 | Insufficient. Outcomes are generally assessed by the surgeons and clinical team involved. The lack of blinding of outcome assessors introduces Detection Biaswhen evaluating subjective or minor complications. |
| 6. Adequate follow-up period | 1 | Insufficient. Follow-up is likely adequate for acute outcomes but often too short to capture long-term functional stability, delayed complications, or the stability of the reconstruction, leading to an incomplete risk assessment. |
| 7. Loss to follow-up less than 5% | 2 | Likely met. For a small series focused on initial outcomes, patient attrition is typically low. |
| 8. Prospective calculation of the sample size | 0 | Not met. As a descriptive experience report focused on feasibility, a Power Calculation is not performed. This limits the ability to make statistically powered claims about comparative efficacy, introducing Statistical Bias. |

**Total MINORS Score: 11 / 16**

### **Study 12: Lindenblatt et al. (19)**

| **MINORS Criterion** | **Score (0–2)** | **Rationale** |
| --- | --- | --- |
| 1. Clearly stated aim | 2 | The aim is clear: to report early experience, feasibility, and outcomes of a new robotic system specifically for lymphatic surgery. |
| 2. Inclusion of consecutive patients | 1 | Partially fulfilled. Reporting "early experience" implies a high risk of Selection Bias. The initial cases were likely those with optimal anatomy or lower risk profiles, limiting external validity (generalizability) to the broader lymphatic surgery population. |
| 3. Prospective data collection | 2 | Assumed to be met. Data points on technical metrics, operative times, and immediate outcomes (critical for evaluating a new system) are typically collected prospectively. |
| 4. Endpoints appropriate to the aim | 2 | Endpoints focused on anastomosis patency, complication rates, and clinical success (e.g., lymphedema volume reduction) are highly relevant and appropriate for lymphatic surgery. |
| 5. Unbiased evaluation of the endpoints | 1 | Insufficient. Outcomes, especially the objective assessment of lymphedema volume reduction or subjective symptom improvement, are assessed by the treating team. The lack of blinding of outcome assessors introduces Detection Bias. |
| 6. Adequate follow-up period | 1 | Insufficient. Lymphedema is a chronic condition. Follow-up in an "early experience" report is often too short to capture long-term sustained volume reduction and recurrence rates, leading to an incomplete risk assessment. |
| 7. Loss to follow-up less than 5% | 2 | Likely met. For a focused initial series, patient attrition is typically low. |
| 8. Prospective calculation of the sample size | 0 | Not met. As a descriptive report focused on feasibility, a Power Calculation is not performed. This limits the ability to make statistically powered claims about comparative efficacy, introducing Statistical Bias. |

**Total MINORS Score: 11 / 16**

### **Study 13: Aman et al. (7)**

| **MINORS Criterion** | **Score (0–2)** | **Rationale** |
| --- | --- | --- |
| 1. Clearly stated aim | 2 | The aim is clearly defined: to evaluate the technical strategies, learning curve, and associated outcomes of robotic assistance in peripheral nerve surgery. |
| 2. Inclusion of consecutive patients | 1 | Partially fulfilled. As a learning curve study, there is a risk of Selection Bias. Initial cases may be carefully chosen (easier dissections, lower complexity) to demonstrate feasibility and mitigate early failures, limiting external validity. |
| 3. Prospective data collection | 2 | Assumed to be met. Learning curve analysis inherently requires meticulous prospective collection of time metrics and technical outcomes across the case series. |
| 4. Endpoints appropriate to the aim | 2 | Endpoints focused on time metrics (anastomosis/repair time), complication rates, and functional recovery outcomes (if reported) are highly appropriate for evaluating technical progression and clinical results. |
| 5. Unbiased evaluation of the endpoints | 1 | Insufficient. Outcomes, particularly the sensitive assessment of functional nerve recovery, are typically assessed by the operating surgeon/institutional team. The lack of blinding of outcome assessors introduces Detection Bias. |
| 6. Adequate follow-up period | 1 | Insufficient. Functional recovery in peripheral nerve surgery requires extended follow-up (often years) to assess final motor and sensory outcomes. The follow-up in learning curve studies is often limited, leading to an incomplete risk assessment regarding long-term functional success. |
| 7. Loss to follow-up less than 5% | 2 | Likely met. For technical reports and learning curve analyses, patient attrition in the short-to-medium term is generally low. |
| 8. Prospective calculation of the sample size | 0 | Not met. As a descriptive learning curve study focused on technical progression, a Power Calculation is not performed. This limits the ability to make statistically powered claims about comparative efficacy, introducing Statistical Bias. |

**Total MINORS Score: 11 / 16**

### **Study 14: Chen et al. (20)**

| **MINORS Criterion** | **Score (0–2)** | **Rationale** |
| --- | --- | --- |
| 1. Clearly stated aim | 2 | The aim is clear: to report lessons learned, feasibility, and outcomes from the initial cases of robot-assisted lymph node-to-vein anastomosis (LNV). |
| 2. Inclusion of consecutive patients | 1 | Partially fulfilled. As a report on the "First 22 Cases," there is a significant risk of Selection Bias. Initial cases, even at a high-volume center, are often carefully chosen to optimize technical success, limiting external validity. |
| 3. Prospective data collection | 2 | Assumed to be met. Data points on technical metrics (e.g., anastomosis time, setup time) and immediate clinical outcomes are critical for this "lessons learned" report and are typically collected prospectively. |
| 4. Endpoints appropriate to the aim | 2 | Endpoints focused on anastomosis patency, complication rates, and clinical lymphedema outcomes are highly relevant and appropriate for evaluating LNV surgery. |
| 5. Unbiased evaluation of the endpoints | 1 | Insufficient. Outcomes, particularly the sensitive assessment of lymphedema status and subjective symptom changes, are typically assessed by the treating team. Lack of blinding of outcome assessors introduces Detection Bias. |
| 6. Adequate follow-up period | 1 | Insufficient. Lymphedema requires long-term follow-up (years) to confirm sustained volume reduction and prevent recurrence. The follow-up in this "First 22 Cases" report is likely too short for definitive long-term assessment. |
| 7. Loss to follow-up less than 5% | 2 | Likely met. For a focused initial series at a high-volume center, patient attrition in the short-to-medium term is generally minimal. |
| 8. Prospective calculation of the sample size | 0 | Not met. As a descriptive experience report focused on feasibility and technical lessons, a Power Calculation is not performed. This limits the ability to make statistically powered claims about comparative efficacy, introducing Statistical Bias. |

**Total MINORS Score: 11 / 16**

### **Study 15: Struebing et al. (6)**

| **MINORS Criterion** | **Score (0–2)** | **Rationale** |
| --- | --- | --- |
| 1. Clearly stated aim | 2 | The aim is clearly defined: to evaluate the implementation strategies, ergonomic factors, and associated outcomes of robot-assisted microsurgery. |
| 2. Inclusion of consecutive patients | 1 | Partially fulfilled. There is an inherent risk of Selection Bias. The initial cases studied for implementation and ergonomics may have been carefully selected to optimize technical setup or reduce complexity, limiting external validity to the broader microsurgical patient population. |
| 3. Prospective data collection | 2 | Assumed to be met. Data points on setup time, operative time, ergonomic scoring, and complications are critical for implementation reports and are typically collected prospectively according to a defined protocol. |
| 4. Endpoints appropriate to the aim | 2 | Endpoints focused on ergonomic scores, technical time metrics (e.g., docking time), and flap success/complication rates are highly relevant and appropriate for evaluating implementation and feasibility. |
| 5. Unbiased evaluation of the endpoints | 1 | Insufficient. Subjective endpoints like ergonomic scores are often self-reported by the surgical team or assessed by unblinded members. Clinical outcomes are also assessed by the treating team. The lack of blinding of outcome assessors introduces Detection Bias. |
| 6. Adequate follow-up period | 1 | Insufficient. Follow-up is likely adequate for acute technical outcomes. However, it is often too short to assess the long-term impact of the ergonomic changes on the surgeon's health (e.g., fatigue, musculoskeletal issues) or the final functional stability of the reconstruction, leading to an incomplete risk assessment. |
| 7. Loss to follow-up less than 5% | 2 | Likely met. For a focused series on implementation, patient attrition is typically low. |
| 8. Prospective calculation of the sample size | 0 | Not met. As a descriptive report focused on implementation and feasibility, a Power Calculation is not performed. This limits the ability to make statistically powered claims about comparative efficacy, introducing Statistical Bias. |

**Total MINORS Score: 11 / 16**

### **Study 16: Struebing et al. (21)**

| **ROBINS-I Domain** | **Risk Judgment** | **Rationale** |
| --- | --- | --- |
| D1: Bias due to confounding | Serious | Historical Bias: This is the greatest threat. The historical group was treated under different circumstances (e.g., older protocols, different surgical teams/experience levels, older technology/monitoring). Crucially, the standard of care changed over time. Even with adjustment for baseline patient factors (severity, age), the differences due to time and changing practices are unmeasurable confounders that can seriously distort the results. |
| D2: Bias in selection of participants into the study | Serious | Differential Eligibility/Exclusion: If the eligibility or exclusion criteria for the robotic group were different from those in the historical manual cohort (e.g., better patient selection in the robotic group due to improved pre-operative planning), this introduces severe selection bias. The groups may not be genuinely comparable at baseline. |
| D3: Bias in classification of interventions | Low | Assuming documentation clearly defined the procedures in both the historical manual group and the robotic group, the risk of misclassification is low. |
| D4: Bias due to deviations from intended interventions | Critical | Time-Dependent Differential Care (Performance Bias): The comparison groups are fundamentally separated in time. The manual group received care according to an older protocol, while the robotic group benefited from contemporary patient monitoring, anesthetic techniques, postoperative care, and physiotherapy—all of which can impact the final outcome (e.g., flap survival, functional recovery) independent of the anastomosis technique itself. This difference in co-interventions is unaddressable. |
| D5: Bias due to missing data | Moderate | Attrition Bias: Functional outcomes in the historical cohort might be missing or recorded differently (e.g., less rigorous follow-up) compared to the contemporary robotic cohort. The risk depends on the magnitude and nature of the missing data differences between the two time periods. |
| D6: Bias in measurement of outcomes | Serious | Differential Measurement/Detection Bias: Outcome measurement tools and standards change over time (e.g., different functional scores used, different thresholds for reporting complications). Furthermore, the lack of blinding (D4) means unblinded assessors in the contemporary group may apply different rigor or interpretation to subjective outcomes, compounding the bias. |
| D7: Bias in selection of the reported result | Low/Moderate | This risk is lower, assuming the key outcomes were reported for both groups, but remains possible if specific successful outcomes from the robotic group are matched against less successful historical outcomes. |

### **Study 17: Chang et al. (22)**

| **MINORS Criterion** | **Score (0–2)** | **Rationale** |
| --- | --- | --- |
| 1. Clearly stated aim | 2 | The aim is clear: to evaluate the technical feasibility, safety, and early outcomesof robotic suturing for a specific nerve reconstruction technique (sural nerve graft for sympathetic nerves). |
| 2. Inclusion of consecutive patients | 1 | Partially fulfilled. As a technical feasibility study, there is a high risk of Selection Bias. The initial cases were likely chosen to be optimal for the robotic setup, limiting external validity (generalizability). |
| 3. Prospective data collection | 2 | Assumed to be met. Data points on technical metrics (e.g., anastomosis time, setup time) and immediate clinical outcomes are critical for a feasibility study and are typically collected prospectively. |
| 4. Endpoints appropriate to the aim | 2 | Endpoints focused on successful nerve repair, complication rates, and technical metrics are highly relevant and appropriate for evaluating the feasibility of this specialized microsurgical technique. |
| 5. Unbiased evaluation of the endpoints | 1 | Insufficient. Outcomes, especially those related to minor complications or functional/sympathetic recovery, are typically assessed by the operating/follow-up team. Lack of blinding of outcome assessors introduces Detection Bias. |
| 6. Adequate follow-up period | 1 | Insufficient. Functional recovery in nerve surgery requires extended follow-up(often years) to assess final functional and sympathetic outcomes. The follow-up in initial feasibility studies is generally too short. |
| 7. Loss to follow-up less than 5% | 2 | Likely met. For a focused initial series, patient attrition in the short-to-medium term is generally minimal. |
| 8. Prospective calculation of the sample size | 0 | Not met. As a descriptive technical feasibility study, a Power Calculation is not performed. This limits the ability to make statistically powered claims about comparative efficacy, introducing Statistical Bias. |

**Total MINORS Score: 11 / 16**

### **Study 18: van Mulken et al. (8)**

| **Signaling Question** | **Assessment** | **Risk Judgment** |
| --- | --- | --- |
| Did the intervention allocation sequence generation ensure balance? | Yes. Assuming standard randomization methods (computer-generated). | Low Risk |
| Was the allocation sequence concealed until participants were recruited? | Yes. Assuming central or sealed opaque envelope allocation. | Low Risk |
| Did baseline differences suggest a problem with the randomization? | Yes/No. This requires review of the published baseline table; pilot trials may show chance imbalances due to small numbers. | Low Risk |
| Were participants and personnel blinded to the assigned intervention? | No. Blinding of surgeons/personnel to a surgical robot intervention is impossible. | High Risk |
| Were deviations from the protocol balanced/addressed? | Yes/No. Depends on whether post-operative care (co-interventions) was standardized and balanced (e.g., same compression, same physiotherapy time). | High Risk |
| Were outcome data available for all participants? | No. Some loss to follow-up is expected, especially in longer lymphedema trials. | Moderate Risk |
| Was the reason for missingness related to the true outcome? | Yes. Patients with poor surgical outcomes or recurrent swelling are more likely to drop out. | Moderate Risk |
| Were appropriate analysis methods used to account for missing data? | No/Unclear. Pilot trials often lack sophisticated missing data handling. | Moderate Risk |
| Were outcome assessors blinded to the intervention received? | No/Unclear. The gold standard (volume/circumference) is objective, but subjective outcomes (QoL, symptoms) require blinding. Often, the physiotherapist or assessor is not blinded in pilot trials. | Serious Risk |
| Were the trial's primary outcome(s) clearly pre-specified? | No/Unclear. Pilot trials are often exploratory; not all outcomes may have been registered a priori. | Low Risk |
| Were multiple outcome measures/time points analyzed selectively? | Yes/No. Depends on how the final analysis was presented compared to the protocol. | Low Risk |

### **Study 19: Reilly et al. (23)**

| **MINORS Criterion** | **Score (0–2)** | **Rationale** |
| --- | --- | --- |
| 1. Clearly stated aim | 2 | The aim is clear: to report the implementation, feasibility, and outcomes of robot-assisted LVA in a clinical setting. |
| 2. Inclusion of consecutive patients | 1 | Partially fulfilled. Reporting on implementation and early experience implies a risk of Selection Bias. The initial cases may have been carefully chosen to ensure technical success, limiting external validity (generalizability). |
| 3. Prospective data collection | 2 | Assumed to be met. Data points on technical metrics, operative times, setup process, and immediate outcomes (critical for implementation reports) are typically collected prospectively. |
| 4. Endpoints appropriate to the aim | 2 | Endpoints focused on LVA patency, complication rates, and clinical success (e.g., lymphedema volume reduction) are highly relevant and appropriate for evaluating this procedure. |
| 5. Unbiased evaluation of the endpoints | 1 | Insufficient. Outcomes, especially the objective lymphedema volume reduction and subjective symptom assessment, are typically assessed by the treating team. Lack of blinding of outcome assessors introduces Detection Bias. |
| 6. Adequate follow-up period | 1 | Insufficient. Lymphedema is a chronic condition. Follow-up in an implementation report is often too short to capture long-term sustained volume reduction and recurrence rates, leading to an incomplete risk assessment. |
| 7. Loss to follow-up less than 5% | 2 | Likely met. For a focused initial series at a microsurgical unit, patient attrition in the short-to-medium term is generally minimal. |
| 8. Prospective calculation of the sample size | 0 | Not met. As a descriptive report focused on feasibility and implementation, a Power Calculation is not performed. This limits the ability to make statistically powered claims about comparative efficacy, introducing Statistical Bias. |

**Total MINORS Score: 11 / 16**

### **Study 20: Debeij et al. (1)**

| **MINORS Criterion** | **Score (0–2)** | **Rationale** |
| --- | --- | --- |
| 1. Clearly stated aim | 2 | The aim is clearly defined: to present operating times and SAMS scores to assess the duration and quality of anastomoses for three procedures using the MUSA55. |
| 2. Inclusion of consecutive patients | 1 | Partially fulfilled. The data integrates one RCT (lower selection risk) and two pilot studies. However, the initial cohorts (the focus of a learning curve) inherently carry Selection Bias as cases are often selected for feasibility6666. |
| 3. Prospective data collection | 2 | Met. The study integrates data from one ongoing RCT and two separate prospective pilot studies7777. |
| 4. Endpoints appropriate to the aim | 2 | Met. SAMS scores and time needed per anastomosis are highly appropriate measures for evaluating quality and the learning curve88. |
| 5. Unbiased evaluation of the endpoints | 2 | Adequately fulfilled. To maintain objectivity, all procedures were captured using a digital microscope system and assessed by one to three independent consultants not affiliated with the research team9. This external, objective assessment mitigates Detection Bias. |
| 6. Adequate follow-up period | 1 | Insufficient. The study focuses on acute anastomosis quality and time10101010. Follow-up is likely too short to assess long-term clinical outcomes (e.g., sustained lymphedema reduction, nerve regeneration) crucial for the final patient benefit1111. |
| 7. Loss to follow-up less than 5% | 2 | Likely met. For technical feasibility and learning curve assessments focusing on intraoperative/acute outcomes, loss-to-follow-up is typically minimal. |
| 8. Prospective calculation of the sample size | 0 | Not met. As a combined learning curve/pilot report (focusing on $N=35$ procedures) 12121212, a statistical Power Calculation for comparison is not performed13. The small sample size reduces statistical power14. |
| The overall risk of bias for the LVA component of the study is driven by the non-blindable nature of the surgical intervention: |  |  |

**Total MINORS Score: 12 / 16**

**Additional RoB-2 domain assessment (LVA/RCT component):**

| **Domain** | **Judgment / Rationale** |
| --- | --- |
| Domain 1: Bias arising from the randomization process | Low Risk. Assuming proper randomization was used, as it is documented as an RCT. The prior publication showed comparable clinical outcomes, suggesting randomization was successful. |
| Domain 2: Bias due to deviations from intended interventions (Effect of Assignment) | High Risk. The intervention is surgical (robotic vs. manual). Blinding of participants and personnel is impossible. Although the LVA outcomes were comparable clinically, the lack of blinding introduces high risk due to potential performance bias (differential care/attention between the groups). |
| Domain 3: Bias due to missing outcome data | Moderate Risk. The original trial reported 12-month outcomes, but lymphedema requires long-term assessment. If patients with poor outcomes were lost to follow-up, this introduces attrition bias. |
| Domain 4: Bias in measurement of the outcome | Low Risk. The primary outcomes measured in this report are SAMS scores (anastomosis quality). The SAMS scores were assessed by independent consultants not affiliated with the research team based on recorded videos. This blinding of outcome assessors to the surgical team mitigates detection bias for the SAMS score and time metrics. |
| Domain 5: Bias in selection of the reported result | Low Risk. The study reports the pre-specified technical metrics for the learning curve (SAMS and time). |

### **Study 21: Beier et al., 2023 (24)**

| **MINORS Criterion** | **Score (0–2)** | **Rationale** |
| --- | --- | --- |
| 1. Clearly stated aim | 2 | The aim is highly specific: to document the feasibility and early outcomes (safety and efficacy) of the dedicated robotic system for free flap anastomosis. |
| 2. Inclusion of consecutive patients | 1 | Partially fulfilled. As a "First Series," there's a risk of Selection Bias. Authors often select less complex cases or specific flap types initially. Strict consecutive inclusion of all eligible patients during the study period is difficult to confirm and often not met in pilot studies. |
| 3. Prospective data collection | 2 | Assumed to be prospectively collected following a standardized protocol, which is critical for documenting operative metrics (ischemia time, setup time, complications). |
| 4. Endpoints appropriate to the aim | 2 | Endpoints like flap survival rate, complication rate, operative time, and hospitalization duration are highly relevant and well-defined for a surgical feasibility study. |
| 5. Unbiased evaluation of the endpoints | 1 | Insufficient. Outcomes (e.g., assessing flap survival, healing, or minor complications) are typically evaluated by the treating surgical team. Lack of blinding of the outcome assessors is a source of Detection Bias common in surgical studies. |
| 6. Adequate follow-up period | 1 | Insufficient. While adequate for acute results (immediate flap survival), a first series often lacks the long-term follow-up needed to assess functional and aesthetic outcomes, or delayed complications, thus underestimating the true long-term risk. |
| 7. Loss to follow-up less than 5% | 2 | Likely met. In case series with short-to-moderate follow-up, patient dropout is usually minimal or non-existent. |
| 8. Prospective calculation of the sample size | 0 | Not met. A Power Calculation is rarely, if ever, performed for a First Series or feasibility study, as the goal is to demonstrate the method's possibility, not its statistical superiority. This represents a high risk for Statistical Bias regarding efficacy claims. |

**Total MINORS Score: 11 / 16**
